# Supplementary material for: From single scenes to extended scenarios: The role of the ventromedial prefrontal cortex in the construction of imagery-rich events
Source: PLoS One. 2026 Feb 12;21(2):e0324764. doi: 10.1371/journal.pone.0324764 (PMC12900345; doi:10.1371/journal.pone.0324764)
Supplement: S1 Table — (DOCX) [file pone.0324764.s001.docx]

S1 Table. Cluster report for the first latent variable from the mean-centered Task-based PLS with the imagery conditions

| Clu# | X | Y | Z | BSR | p | Size (voxels) | aal.distance | aal.label | ba.distance | ba.label |
| --- | --- | --- | --- | --- | --- | --- | --- | --- | --- | --- |
| 1 | 13,5 | -49,5 | 1,8 | 8,30 | 0,0000 | 22052 | 0 | Lingual_R | 0 | Right-VisualAssoc (18) |
| 2 | -15,3 | -39,6 | -14,4 | 7,26 | 0,0000 | 31767 | 0 | Cerebelum_4_5_L | 1 | Left-Fusiform (37) |
| 3 | 17,1 | -54,9 | 14,4 | 6,95 | 0,0000 | 1885 | 0 | Calcarine_R | 0 | Right-BA23 |
| 4 | 45,9 | -40,5 | -1,8 | 6,83 | 0,0000 | 1881 | 2,45 | Temporal_Mid_R | 0 | Right-BA21 |
| 5 | -5,4 | -85,5 | -13,5 | 6,67 | 0,0000 | 2324 | 0 | Calcarine_L | 0 | Left-VisualAssoc (18) |
| 6 | -35,1 | -89,1 | 5,4 | 6,20 | 0,0000 | 1076 | 0 | Occipital_Mid_L | 0 | Left-VisualAssoc (18) |
| 7 | 15,3 | 27,9 | -11,7 | 5,62 | 0,0000 | 5787 | 0 | Rectus_R | 1 | Right-BA11 |
| 8 | -17,1 | -79,2 | -35,1 | 5,31 | 0,0000 | 1696 | 0 | Cerebelum_Crus2_L | 17,23 | Left-VisualAssoc (18) |
| 9 | 39,6 | 24,3 | -19,8 | 5,31 | 0,0000 | 158 | 0 | Temporal_Pole_Sup_R | 0 | Right-BA47 |
| 10 | 40,5 | -81,9 | 28,8 | 5,16 | 0,0000 | 2223 | 0 | Occipital_Mid_R | 0 | Right-BA19 |
| 11 | 51,3 | -8,1 | -18,9 | 5,04 | 0,0000 | 2142 | 0 | Temporal_Mid_R | 0 | Right-BA21 |
| 12 | -41,4 | -19,8 | -29,7 | 4,90 | 0,0000 | 326 | 0 | Temporal_Inf_L | 0 | Left-BA20 |
| 13 | 0 | 5,4 | -6,3 | 4,62 | 0,0000 | 2144 | 2,24 | Olfactory_L | 2,82 | Right-BA25 |
| 14 | -10,8 | -8,1 | 23,4 | 4,59 | 0,0000 | 1308 | 2,45 | Caudate_L | 3,60 | Left-Caudate (48) |
| 15 | 27 | 36,9 | 41,4 | 4,57 | 0,0000 | 1838 | 0 | Frontal_Mid_R | 0 | Right-BA9 |
| 16 | -27 | 43,2 | 18,9 | 4,57 | 0,0000 | 3484 | 0 | Frontal_Mid_L | 0 | Left-BA10 |
| 17 | -66,6 | -16,2 | -6,3 | 4,44 | 0,0000 | 275 | 0 | Temporal_Mid_L | 0 | Left-BA21 |
| 18 | -9,9 | 36,9 | -13,5 | 4,41 | 0,0000 | 1075 | 0 | Rectus_L | 0 | Left-BA11 |
| 19 | -47,7 | -76,5 | 18 | 4,37 | 0,0000 | 533 | 0 | Occipital_Mid_L | 0 | Left-BA19 |
| 20 | -27 | -81,9 | 26,1 | 4,34 | 0,0000 | 3425 | 0 | Occipital_Mid_L | 0 | Left-BA19 |
| 21 | -25,2 | 2,7 | 62,1 | 4,31 | 0,0000 | 745 | 0 | Frontal_Mid_L | 0 | Left-BA6 |
| 22 | -49,5 | 7,2 | -18,9 | 4,12 | 0,0000 | 896 | 0 | Temporal_Pole_Sup_L | 0 | Left-BA38 |
| 23 | -46,8 | 32,4 | -10,8 | 4,11 | 0,0000 | 1987 | 0 | Frontal_Inf_Orb_L | 0 | Left-BA47 |
| 24 | -19,8 | 38,7 | 34,2 | 3,96 | 0,0001 | 1343 | 0 | Frontal_Sup_L | 0 | Left-BA9 |
| 25 | -54 | 27,9 | 12,6 | 3,95 | 0,0001 | 673 | 0 | Frontal_Inf_Tri_L | 0 | Left-BA45 |
| 26 | -2,7 | 0 | 12,6 | 3,79 | 0,0002 | 278 | 3,16 | Caudate_L | 5,38 | Left-Thalamus (50) |
| 27 | -21,6 | -18 | -5,4 | 3,67 | 0,0002 | 460 | 4 | Thalamus_L | 7,34 | Left-Thalamus (50) |
| 28 | 45 | -74,7 | -3,6 | 3,67 | 0,0002 | 161 | 0 | Occipital_Inf_R | 0 | Right-BA19 |
| 29 | 27,9 | 51,3 | 28,8 | 3,62 | 0,0003 | 243 | 0 | Frontal_Mid_R | 0 | Right-BA9 |
| 30 | -4,5 | 56,7 | -14,4 | 3,58 | 0,0003 | 1596 | 0 | Rectus_L | 0 | Left-BA10 |
| 31 | 28,8 | -72,9 | -37,8 | 3,58 | 0,0003 | 171 | 0 | Cerebelum_Crus2_R | 18,46 | Right-Fusiform (37) |
| 32 | -49,5 | 18 | -9,9 | 3,54 | 0,0004 | 146 | 0 | Temporal_Pole_Sup_L | 0 | Left-BA47 |
| 33 | 32,4 | -56,7 | -15,3 | 3,54 | 0,0004 | 455 | 0 | Fusiform_R | 0 | Right-Fusiform (37) |
| 34 | -20,7 | 18,9 | 40,5 | 3,44 | 0,0006 | 428 | 1,41 | Frontal_Mid_L | 0 | Left-BA8 |
| 35 | -59,4 | -12,6 | -14,4 | 3,42 | 0,0006 | 158 | 0 | Temporal_Mid_L | 0 | Left-BA21 |
| 36 | -9,9 | 9 | 47,7 | 3,32 | 0,0009 | 450 | 0 | Supp_Motor_Area_L | 2,44 | Left-BA6 |
| 37 | -40,5 | -77,4 | -8,1 | 3,27 | 0,0011 | 230 | 0 | Occipital_Inf_L | 0 | Left-BA19 |
| 38 | 9 | 49,5 | -14,4 | 3,21 | 0,0013 | 295 | 0 | Rectus_R | 0 | Right-BA11 |
| 39 | -30,6 | -0,9 | 36,9 | 3,14 | 0,0017 | 1543 | 5 | Precentral_L | 4,12 | Left-BA6 |
| 40 | 60,3 | 5,4 | 8,1 | 3,13 | 0,0017 | 112 | 0 | Rolandic_Oper_R | 0 | Right-BA6 |
| 41 | 13,5 | -77,4 | -30,6 | 3,11 | 0,0019 | 716 | 0 | Cerebelum_Crus1_R | 15,81 | Right-VisualAssoc (18) |
| 42 | -18 | -72 | 45,9 | 3,08 | 0,0021 | 404 | 0 | Parietal_Sup_L | 0 | Left-BA7 |
| 43 | 26,1 | 3,6 | 20,7 | 3,04 | 0,0024 | 261 | 4 | Caudate_R | 6,08 | Right-Caudate (48) |
| 44 | 22,5 | 63 | 16,2 | 2,85 | 0,0044 | 124 | 0 | Frontal_Sup_R | 0 | Right-BA10 |
| 45 | 0 | -40,5 | 7,2 | 2,82 | 0,0049 | 111 | 2,23 | Cingulum_Post_R | 2,23 | Right-BA30 |
| 46 | 0,9 | 58,5 | 15,3 | 2,77 | 0,0057 | 223 | 0 | Frontal_Sup_Medial_R | 0 | Right-BA10 |
| 47 | -41,4 | -63 | -40,5 | 2,76 | 0,0057 | 170 | 0 | Cerebelum_Crus2_L | 16,88 | Left-Fusiform (37) |
| 48 | -17,1 | 62,1 | 16,2 | 2,75 | 0,0060 | 149 | 0 | Frontal_Sup_L | 0 | Left-BA10 |
| 49 | -45,9 | -24,3 | 25,2 | 2,74 | 0,0061 | 222 | 1 | Rolandic_Oper_L | 1,73 | Left-PrimSensory (1) |
| 50 | 1,8 | -66,6 | 35,1 | 2,66 | 0,0078 | 377 | 0 | Precuneus_R | 0 | Right-BA31 |
| 51 | 61,2 | 18 | 29,7 | -6,13 | 0,0000 | 1336 | 0 | Frontal_Inf_Oper_R | 0 | Right-BA44 |
| 52 | 64,8 | -31,5 | 43,2 | -6,12 | 0,0000 | 74358 | 0 | SupraMarginal_R | 1,41 | Right-BA40 |
| 53 | -59,4 | -10,8 | 5,4 | -5,72 | 0,0000 | 2837 | 0 | Temporal_Sup_L | 0 | Left-PrimAuditory (41) |
| 54 | -40,5 | -9 | 9,9 | -5,11 | 0,0000 | 3412 | 0 | Insula_L | 0 | Left-PrimMotor (4) |
| 55 | 56,7 | -53,1 | 38,7 | -4,59 | 0,0000 | 1452 | 0 | Parietal_Inf_R | 0 | Right-BA39 |
| 56 | -8,1 | -94,5 | 11,7 | -4,35 | 0,0000 | 5559 | 0 | Calcarine_L | 0 | Left-VisualAssoc (18) |
| 57 | 60,3 | -58,5 | 0,9 | -4,29 | 0,0000 | 633 | 0 | Temporal_Mid_R | 0 | Right-Fusiform (37) |
| 58 | 8,1 | -22,5 | 66,6 | -4,15 | 0,0000 | 971 | 0 | Paracentral_Lobule_R | 0 | Right-BA6 |
| 59 | -27,9 | -50,4 | 64,8 | -4,14 | 0,0000 | 303 | 0 | Parietal_Sup_L | 0 | Left-BA7 |
| 60 | 13,5 | -90,9 | 29,7 | -4,02 | 0,0001 | 291 | 0 | Cuneus_R | 0 | Right-BA19 |
| 61 | 17,1 | 54 | 20,7 | -3,91 | 0,0001 | 367 | 0 | Frontal_Sup_R | 0 | Right-BA10 |
| 62 | 17,1 | 30,6 | -21,6 | -3,86 | 0,0001 | 116 | 0 | Frontal_Sup_Orb_R | 0 | Right-BA11 |
| 63 | 63,9 | -18 | 12,6 | -3,84 | 0,0001 | 1782 | 0 | Temporal_Sup_R | 0 | Right-BA40 |
| 64 | 9,9 | -27 | -41,4 | -3,80 | 0,0001 | 328 | 9,89 | Cerebelum_10_R | 19,64 | Right-Parahip (36) |
| 65 | -50,4 | -65,7 | -8,1 | -3,79 | 0,0001 | 759 | 0 | Temporal_Inf_L | 0 | Left-Fusiform (37) |
| 66 | -47,7 | 40,5 | 9,9 | -3,69 | 0,0002 | 176 | 0 | Frontal_Inf_Tri_L | 0 | Left-BA46 |
| 67 | 46,8 | -43,2 | -14,4 | -3,53 | 0,0004 | 719 | 0 | Temporal_Inf_R | 0 | Right-Fusiform (37) |
| 68 | -16,2 | 47,7 | 18 | -3,53 | 0,0004 | 1908 | 2,23 | Frontal_Sup_L | 3 | Left-BA10 |
| 69 | 49,5 | 21,6 | 43,2 | -3,49 | 0,0005 | 279 | 0 | Frontal_Mid_R | 1 | Right-BA8 |
| 70 | 3,6 | -72,9 | -40,5 | -3,49 | 0,0005 | 661 | 0 | Cerebelum_7b_R | 26,11 | Right-VisualAssoc (18) |
| 71 | -57,6 | 12,6 | 36 | -3,36 | 0,0008 | 237 | 0 | Precentral_L | 1 | Left-BA6 |
| 72 | -6,3 | -37,8 | -36 | -3,29 | 0,0010 | 317 | 2,23 | Vermis_10 | 20,34 | Left-Fusiform (37) |
| 73 | -29,7 | 26,1 | 10,8 | -3,27 | 0,0011 | 174 | 0 | Insula_L | 0 | Left-BA45 |
| 74 | 50,4 | -52,2 | -6,3 | -3,25 | 0,0012 | 1162 | 0 | Temporal_Inf_R | 0 | Right-Fusiform (37) |
| 75 | -17,1 | -0,9 | 69,3 | -3,20 | 0,0014 | 162 | 0 | Frontal_Sup_L | 0 | Left-BA6 |
| 76 | -45 | 18,9 | 35,1 | -3,18 | 0,0015 | 394 | 0 | Frontal_Inf_Oper_L | 0 | Left-BA8 |
| 77 | 47,7 | -27 | -13,5 | -3,12 | 0,0018 | 131 | 3 | Fusiform_R | 1 | Right-BA20 |
| 78 | 22,5 | -5,4 | 69,3 | -3,10 | 0,0019 | 123 | 0 | Frontal_Sup_R | 0 | Right-BA6 |
| 79 | -26,1 | -27 | 68,4 | -3,08 | 0,0021 | 191 | 0 | Postcentral_L | 0 | Left-PrimMotor (4) |
| 80 | 8,1 | -2,7 | 61,2 | -3,05 | 0,0023 | 104 | 0 | Supp_Motor_Area_R | 0 | Right-BA6 |
| 81 | 36 | -8,1 | -32,4 | -3,02 | 0,0025 | 363 | 0 | Fusiform_R | 0 | Right-Parahip (36) |
| 82 | -10,8 | 13,5 | 25,2 | -2,98 | 0,0029 | 942 | 3 | Cingulum_Ant_L | 4 | Left-BA24 |
| 83 | -41,4 | 5,4 | 24,3 | -2,90 | 0,0037 | 125 | 0 | Frontal_Inf_Oper_L | 0 | Left-BA44 |
| 84 | 18,9 | -78,3 | 10,8 | -2,61 | 0,0092 | 130 | 0 | Calcarine_R | 0 | Right-PrimVisual (17) |
| 85 | 24,3 | -35,1 | 63 | -2,60 | 0,0093 | 176 | 0 | Postcentral_R | 0 | Right-PrimSensory (1) |
