# Supplementary material for: From single scenes to extended scenarios: The role of the ventromedial prefrontal cortex in the construction of imagery-rich events
Source: PLoS One. 2026 Feb 12;21(2):e0324764. doi: 10.1371/journal.pone.0324764 (PMC12900345; doi:10.1371/journal.pone.0324764)
Supplement: S2 Table — (DOCX) [file pone.0324764.s002.docx]

S2 Table. Cluster report for the second latent variable from the mean-centered Task-based PLS with the imagery conditions

| Clu# | X | Y | Z | BSR | p | Size (voxels) | aal.distance | aal.label | ba.distance | ba.label |
| --- | --- | --- | --- | --- | --- | --- | --- | --- | --- | --- |
| 1 | -43,2 | -65,7 | 28,8 | 7,81 | 0,0000 | 6757 | 0,00 | Angular_L | 0,00 | Left-BA39 |
| 2 | 52,2 | -68,4 | 27,9 | 7,40 | 0,0000 | 4098 | 0,00 | Occipital_Mid_R | 0,00 | Right-BA39 |
| 3 | 55,8 | 36 | -2,7 | 6,55 | 0,0000 | 2535 | 0,00 | Frontal_Inf_Orb_R | 0,00 | Right-BA47 |
| 4 | -2,7 | -48,6 | 26,1 | 6,45 | 0,0000 | 32076 | 0,00 | Cingulum_Post_L | 0,00 | Left-BA23 |
| 5 | -8,1 | 59,4 | 31,5 | 6,16 | 0,0000 | 11178 | 0,00 | Frontal_Sup_Medial_L | 0,00 | Left-BA9 |
| 6 | -3,6 | 54 | -9 | 6,04 | 0,0000 | 8555 | 0,00 | Frontal_Med_Orb_L | 0,00 | Left-BA10 |
| 7 | 6,3 | 13,5 | 27 | 5,65 | 0,0000 | 7742 | 0,00 | Cingulum_Ant_R | 0,00 | Right-BA24 |
| 8 | -54 | 31,5 | 6,3 | 4,74 | 0,0000 | 1163 | 0,00 | Frontal_Inf_Tri_L | 0,00 | Left-BA45 |
| 9 | -54 | 11,7 | -31,5 | 4,56 | 0,0000 | 528 | 0,00 | Temporal_Pole_Mid_L | 0,00 | Left-BA38 |
| 10 | 44,1 | -18,9 | 62,1 | 4,37 | 0,0000 | 1688 | 0,00 | Precentral_R | 0,00 | Right-PrimMotor (4) |
| 11 | -65,7 | -12,6 | -10,8 | 4,32 | 0,0000 | 1517 | 0,00 | Temporal_Mid_L | 0,00 | Left-BA21 |
| 12 | -20,7 | -46,8 | -31,5 | 4,15 | 0,0000 | 381 | 4,24 | Cerebelum_6_L | 12,33 | Left-Fusiform (37) |
| 13 | -3,6 | -74,7 | -35,1 | 4,13 | 0,0000 | 1126 | 0,00 | Cerebelum_Crus2_L | 21,31 | Left-VisualAssoc (18) |
| 14 | -63,9 | -36 | -16,2 | 3,98 | 0,0001 | 281 | 0,00 | Temporal_Inf_L | 0,00 | Left-BA21 |
| 15 | 14,4 | -8,1 | 59,4 | 3,96 | 0,0001 | 6726 | 0,00 | Supp_Motor_Area_R | 2,24 | Right-BA6 |
| 16 | -25,2 | 24,3 | 37,8 | 3,94 | 0,0001 | 1426 | 0,00 | Frontal_Mid_L | 0,00 | Left-BA9 |
| 17 | -4,5 | 12,6 | 65,7 | 3,92 | 0,0001 | 1026 | 0,00 | Supp_Motor_Area_L | 0,00 | Left-BA6 |
| 18 | -38,7 | 54,9 | -3,6 | 3,91 | 0,0001 | 163 | 0,00 | Frontal_Mid_Orb_L | 0,00 | Left-BA10 |
| 19 | -39,6 | 9 | -35,1 | 3,85 | 0,0001 | 236 | 0,00 | Temporal_Inf_L | 0,00 | Left-BA38 |
| 20 | 26,1 | 30,6 | 42,3 | 3,60 | 0,0003 | 1992 | 0,00 | Frontal_Mid_R | 0,00 | Right-BA8 |
| 21 | 22,5 | -19,8 | -13,5 | 3,56 | 0,0004 | 257 | 0,00 | Hippocampus_R | 1,00 | Right-Parahip (36) |
| 22 | 25,2 | -81 | -33,3 | 3,53 | 0,0004 | 1838 | 0,00 | Cerebelum_Crus1_R | 15,00 | Right-BA19 |
| 23 | 52,2 | 15,3 | -27,9 | 3,49 | 0,0005 | 364 | 0,00 | Temporal_Pole_Mid_R | 0,00 | Right-BA38 |
| 24 | -18 | -38,7 | 19,8 | 3,47 | 0,0005 | 430 | 6,40 | Cingulum_Post_L | 7,87 | Left-BA23 |
| 25 | -3,6 | 25,2 | -9,9 | 3,38 | 0,0007 | 1348 | 0,00 | Olfactory_L | 0,00 | Left-BA32 |
| 26 | -37,8 | -33,3 | -3,6 | 3,37 | 0,0007 | 258 | 1,00 | Hippocampus_L | 2,00 | Left-Caudate (48) |
| 27 | 28,8 | 4,5 | 19,8 | 3,36 | 0,0008 | 495 | 4,00 | Putamen_R | 5,83 | Right-BA44 |
| 28 | -56,7 | 31,5 | -23,4 | 3,21 | 0,0013 | 144 | 10,00 | Frontal_Inf_Orb_L | 9,90 | Left-BA47 |
| 29 | 42,3 | 9 | -13,5 | 3,17 | 0,0015 | 404 | 1,00 | Insula_R | 1,00 | Right-Insula (13) |
| 30 | -57,6 | -63 | 15,3 | 3,14 | 0,0017 | 196 | 0,00 | Temporal_Mid_L | 0,00 | Left-BA39 |
| 31 | -16,2 | -95,4 | 10,8 | 3,11 | 0,0019 | 688 | 0,00 | Occipital_Sup_L | 0,00 | Left-VisualAssoc (18) |
| 32 | -9,9 | -71,1 | -6,3 | 3,06 | 0,0022 | 494 | 0,00 | Lingual_L | 0,00 | Left-VisualAssoc (18) |
| 33 | 7,2 | -58,5 | -50,4 | 3,05 | 0,0023 | 447 | 0,00 | Cerebelum_9_R | 36,69 | Right-Fusiform (37) |
| 34 | -51,3 | -51,3 | 0,9 | 3,03 | 0,0025 | 334 | 0,00 | Temporal_Mid_L | 0,00 | Left-Fusiform (37) |
| 35 | -32,4 | 7,2 | 18 | 3,02 | 0,0025 | 560 | 0,00 | Insula_L | 1,00 | Left-BA44 |
| 36 | 31,5 | -30,6 | -6,3 | 2,99 | 0,0028 | 298 | 0,00 | Hippocampus_R | 0,00 | Right-Caudate (48) |
| 37 | 66,6 | -38,7 | 6,3 | 2,99 | 0,0028 | 419 | 0,00 | Temporal_Mid_R | 0,00 | Right-BA21 |
| 38 | -8,1 | -88,2 | -4,5 | 2,98 | 0,0028 | 171 | 0,00 | Calcarine_L | 0,00 | Left-VisualAssoc (18) |
| 39 | -35,1 | -63,9 | -48,6 | 2,94 | 0,0033 | 209 | 0,00 | Cerebelum_7b_L | 27,04 | Left-Fusiform (37) |
| 40 | 0 | -14,4 | 9,9 | 2,90 | 0,0038 | 109 | 0,00 | Thalamus_R | 0,00 | Right-Thalamus (50) |
| 41 | -45,9 | -19,8 | 49,5 | 2,88 | 0,0040 | 179 | 0,00 | Postcentral_L | 0,00 | Left-PrimSensory (1) |
| 42 | -18,9 | -87,3 | -35,1 | 2,86 | 0,0043 | 106 | 0,00 | Cerebelum_Crus2_L | 14,14 | Left-VisualAssoc (18) |
| 43 | 42,3 | -26,1 | 18 | 2,84 | 0,0045 | 160 | 0,00 | Rolandic_Oper_R | 0,00 | Right-BA40 |
| 44 | -22,5 | -4,5 | 15,3 | 2,83 | 0,0046 | 128 | 1,73 | Putamen_L | 3,46 | Left-Putamen (49) |
| 45 | 36,9 | 44,1 | 19,8 | 2,77 | 0,0056 | 155 | 0,00 | Frontal_Mid_R | 0,00 | Right-BA10 |
| 46 | 43,2 | -21,6 | -0,9 | 2,76 | 0,0058 | 575 | 0,00 | Temporal_Sup_R | 0,00 | Right-PrimAuditory (41) |
| 47 | 0,9 | -29,7 | -18 | 2,73 | 0,0063 | 109 | 4,00 | Vermis_1_2 | 12,96 | Right-Parahip (36) |
| 48 | -51,3 | -38,7 | 47,7 | -8,33 | 0,0000 | 23397 | 0,00 | Parietal_Inf_L | 0,00 | Left-BA40 |
| 49 | 27 | -66,6 | 45 | -7,53 | 0,0000 | 29883 | 0,00 | Occipital_Sup_R | 0,00 | Right-BA39 |
| 50 | 23,4 | 33,3 | -17,1 | -6,44 | 0,0000 | 1774 | 0,00 | Frontal_Sup_Orb_R | 0,00 | Right-BA11 |
| 51 | -54,9 | -65,7 | -9,9 | -6,39 | 0,0000 | 11729 | 0,00 | Temporal_Inf_L | 0,00 | Left-Fusiform (37) |
| 52 | 0,9 | 15,3 | 6,3 | -6,37 | 0,0000 | 11096 | 4,00 | Caudate_R | 5,10 | Right-Caudate (48) |
| 53 | -20,7 | -72 | 38,7 | -6,06 | 0,0000 | 6481 | 0,00 | Occipital_Sup_L | 0,00 | Left-BA7 |
| 54 | -49,5 | 43,2 | 13,5 | -5,52 | 0,0000 | 1227 | 0,00 | Frontal_Inf_Tri_L | 1,41 | Left-BA46 |
| 55 | -25,2 | -19,8 | -2,7 | -5,49 | 0,0000 | 1834 | 2,83 | Thalamus_L | 6,16 | Left-Putamen (49) |
| 56 | -55,8 | 12,6 | 33,3 | -5,33 | 0,0000 | 7817 | 0,00 | Precentral_L | 1,00 | Left-BA6 |
| 57 | 47,7 | 41,4 | 27 | -5,21 | 0,0000 | 4332 | 0,00 | Frontal_Mid_R | 2,00 | Right-BA9 |
| 58 | 14,4 | 18 | 38,7 | -5,17 | 0,0000 | 10111 | 0,00 | Cingulum_Mid_R | 2,00 | Right-BA8 |
| 59 | 64,8 | 20,7 | 23,4 | -4,95 | 0,0000 | 1330 | 1,00 | Frontal_Inf_Oper_R | 2,24 | Right-BA44 |
| 60 | 44,1 | 9 | 27,9 | -4,85 | 0,0000 | 2810 | 0,00 | Frontal_Inf_Oper_R | 0,00 | Right-BA44 |
| 61 | 54 | -52,2 | -24,3 | -4,78 | 0,0000 | 780 | 0,00 | Temporal_Inf_R | 0,00 | Right-Fusiform (37) |
| 62 | 51,3 | -68,4 | -16,2 | -4,73 | 0,0000 | 13362 | 1,00 | Fusiform_R | 0,00 | Right-Fusiform (37) |
| 63 | -46,8 | 36,9 | 26,1 | -4,73 | 0,0000 | 749 | 0,00 | Frontal_Inf_Tri_L | 0,00 | Left-BA9 |
| 64 | 26,1 | -66,6 | 24,3 | -4,63 | 0,0000 | 210 | 0,00 | Occipital_Sup_R | 2,24 | Right-BA19 |
| 65 | 20,7 | 2,7 | 70,2 | -4,60 | 0,0000 | 1180 | 0,00 | Frontal_Sup_R | 1,00 | Right-BA6 |
| 66 | 31,5 | -44,1 | 5,4 | -4,30 | 0,0000 | 1458 | 5,00 | Precuneus_R | 5,20 | Right-Hippocampus (54) |
| 67 | -28,8 | -83,7 | 20,7 | -4,19 | 0,0000 | 1209 | 0,00 | Occipital_Mid_L | 0,00 | Left-BA19 |
| 68 | -22,5 | 43,2 | -18,9 | -4,10 | 0,0000 | 1336 | 0,00 | Frontal_Mid_Orb_L | 0,00 | Left-BA11 |
| 69 | 3,6 | 1,8 | 31,5 | -3,92 | 0,0001 | 810 | 0,00 | Cingulum_Mid_R | 0,00 | Right-BA24 |
| 70 | 34,2 | -6,3 | -28,8 | -3,91 | 0,0001 | 320 | 0,00 | Fusiform_R | 2,00 | Right-Parahip (36) |
| 71 | -41,4 | -0,9 | -24,3 | -3,90 | 0,0001 | 987 | 2,00 | Temporal_Mid_L | 3,61 | Left-BA38 |
| 72 | 68,4 | -29,7 | 2,7 | -3,87 | 0,0001 | 238 | 0,00 | Temporal_Sup_R | 0,00 | Right-BA22 |
| 73 | -16,2 | -99,9 | -9 | -3,79 | 0,0002 | 192 | 0,00 | Occipital_Inf_L | 0,00 | Left-VisualAssoc (18) |
| 74 | 23,4 | -91,8 | -6,3 | -3,75 | 0,0002 | 1647 | 0,00 | Lingual_R | 0,00 | Right-VisualAssoc (18) |
| 75 | 54 | -15,3 | 29,7 | -3,72 | 0,0002 | 950 | 0,00 | SupraMarginal_R | 0,00 | Right-PrimSensory (1) |
| 76 | -53,1 | 1,8 | 46,8 | -3,71 | 0,0002 | 217 | 0,00 | Precentral_L | 0,00 | Left-BA6 |
| 77 | -27 | 51,3 | 2,7 | -3,68 | 0,0002 | 390 | 0,00 | Frontal_Mid_L | 0,00 | Left-BA10 |
| 78 | 54,9 | 27,9 | -34,2 | -3,66 | 0,0003 | 319 | 5,74 | Temporal_Pole_Mid_R | 8,06 | Right-BA38 |
| 79 | -41,4 | -14,4 | -18 | -3,60 | 0,0003 | 208 | 2,00 | Temporal_Inf_L | 4,47 | Left-BA20 |
| 80 | -63 | -20,7 | 6,3 | -3,57 | 0,0004 | 293 | 0,00 | Temporal_Sup_L | 0,00 | Left-PrimAuditory (41) |
| 81 | -35,1 | -72,9 | -31,5 | -3,52 | 0,0004 | 547 | 0,00 | Cerebelum_Crus1_L | 12,69 | Left-BA19 |
| 82 | -43,2 | 7,2 | 0,9 | -3,51 | 0,0005 | 1296 | 0,00 | Insula_L | 0,00 | Left-Insula (13) |
| 83 | 63 | -45,9 | 29,7 | -3,30 | 0,0010 | 231 | 0,00 | SupraMarginal_R | 0,00 | Right-BA39 |
| 84 | 63,9 | -6,3 | 3,6 | -3,26 | 0,0011 | 252 | 0,00 | Temporal_Sup_R | 0,00 | Right-PrimAuditory (41) |
| 85 | 4,5 | -22,5 | 18 | -3,26 | 0,0011 | 2040 | 3,61 | Thalamus_R | 5,39 | Right-Thalamus (50) |
| 86 | 6,3 | -24,3 | -33,3 | -3,25 | 0,0012 | 346 | 11,36 | Cerebelum_3_R | 17,55 | Right-Parahip (36) |
| 87 | -4,5 | -27,9 | -6,3 | -3,17 | 0,0015 | 610 | 6,08 | Vermis_3 | 4,12 | Left-Thalamus (50) |
| 88 | 26,1 | -19,8 | -25,2 | -3,13 | 0,0018 | 494 | 0,00 | ParaHippocampal_R | 0,00 | Right-Parahip (36) |
| 89 | 20,7 | -13,5 | -3,6 | -3,11 | 0,0019 | 423 | 3,00 | Thalamus_R | 5,92 | Right-Thalamus (50) |
| 90 | -64,8 | -4,5 | 10,8 | -3,03 | 0,0024 | 372 | 0,00 | Rolandic_Oper_L | 0,00 | Left-PrimMotor (4) |
| 91 | 38,7 | 1,8 | 3,6 | -2,93 | 0,0034 | 380 | 0,00 | Insula_R | 0,00 | Right-Insula (13) |
| 92 | -21,6 | -16,2 | 70,2 | -2,87 | 0,0042 | 566 | 0,00 | Precentral_L | 0,00 | Left-BA6 |
| 93 | -38,7 | -44,1 | 16,2 | -2,85 | 0,0043 | 533 | 4,24 | Temporal_Sup_L | 4,58 | Left-BA22 |
| 94 | -20,7 | 8,1 | -20,7 | -2,80 | 0,0051 | 114 | 0,00 | Temporal_Pole_Sup_L | 2,83 | Left-Amygdala (53) |
| 95 | 69,3 | -20,7 | 16,2 | -2,72 | 0,0066 | 139 | 0,00 | Temporal_Sup_R | 0,00 | Right-BA40 |
| 96 | -6,3 | -72,9 | -19,8 | -2,69 | 0,0070 | 201 | 0,00 | Cerebelum_Crus1_L | 9,00 | Left-VisualAssoc (18) |
| 97 | 18 | 64,8 | 9 | -2,65 | 0,0080 | 121 | 0,00 | Frontal_Sup_R | 0,00 | Right-BA10 |
| 98 | -12,6 | -18,9 | 0,9 | -2,65 | 0,0080 | 278 | 0,00 | Thalamus_L | 0,00 | Left-Thalamus (50) |
| 99 | -12,6 | -102,6 | 0,9 | -2,64 | 0,0084 | 283 | 0,00 | Occipital_Mid_L | 0,00 | Left-VisualAssoc (18) |
| 100 | 54,9 | -13,5 | -5,4 | -2,59 | 0,0097 | 113 | 0,00 | Temporal_Sup_R | 0,00 | Right-BA22 |
| 101 | -27,9 | -29,7 | -27,9 | -2,55 | 0,0107 | 127 | 0,00 | Cerebelum_4_5_L | 1,00 | Left-Fusiform (37) |
