# Supplementary material for: From single scenes to extended scenarios: The role of the ventromedial prefrontal cortex in the construction of imagery-rich events
Source: PLoS One. 2026 Feb 12;21(2):e0324764. doi: 10.1371/journal.pone.0324764 (PMC12900345; doi:10.1371/journal.pone.0324764)
Supplement: S3 Table — (DOCX) [file pone.0324764.s003.docx]

S3 Table. Cluster report from the first latent variable from the mean centered task-based Seed PLS

| Clu# | X | Y | Z | BSR | p | Size (voxels) | aal.distance | aal.label | ba.distance | ba.label |
| --- | --- | --- | --- | --- | --- | --- | --- | --- | --- | --- |
| 1 | 0,9 | 56,7 | -12,6 | 16,09 | 0,0000 | 67741 | 0,00 | Frontal_Med_Orb_R | 1,41 | Right-BA10 |
| 2 | 2,7 | -15,3 | -13,5 | 11,83 | 0,0000 | 118208 | 12,81 | Hippocampus_R | 8,25 | Right-Hypothalamus (55) |
| 3 | -64,8 | -26,1 | 18,9 | 10,33 | 0,0000 | 4112 | 0,00 | Temporal_Sup_L | 0,00 | Left-BA40 |
| 4 | -25,2 | 27,9 | 41,4 | 9,18 | 0,0000 | 24175 | 0,00 | Frontal_Mid_L | 0,00 | Left-BA9 |
| 5 | 54,9 | 42,3 | -17,1 | 8,77 | 0,0000 | 3253 | 3,46 | Frontal_Inf_Orb_R | 3,00 | Right-BA47 |
| 6 | -38,7 | -69,3 | 29,7 | 8,44 | 0,0000 | 75921 | 0,00 | Occipital_Mid_L | 0,00 | Left-BA39 |
| 7 | -65,7 | 9 | -8,1 | 7,77 | 0,0000 | 20055 | 5,83 | Temporal_Sup_L | 5,39 | Left-BA38 |
| 8 | -28,8 | 58,5 | 7,2 | 7,77 | 0,0000 | 11174 | 0,00 | Frontal_Mid_L | 0,00 | Left-BA10 |
| 9 | -57,6 | 23,4 | 8,1 | 7,45 | 0,0000 | 1476 | 0,00 | Frontal_Inf_Tri_L | 0,00 | Left-BA45 |
| 10 | -52,2 | -15,3 | 30,6 | 7,03 | 0,0000 | 17259 | 0,00 | Postcentral_L | 0,00 | Left-PrimMotor (4) |
| 11 | 54,9 | -46,8 | 28,8 | 6,95 | 0,0000 | 26694 | 0,00 | SupraMarginal_R | 0,00 | Right-BA39 |
| 12 | -60,3 | 33,3 | -4,5 | 6,34 | 0,0000 | 372 | 4,24 | Frontal_Inf_Tri_L | 3,32 | Left-BA47 |
| 13 | 2,7 | -69,3 | -27 | 5,59 | 0,0000 | 9821 | 0,00 | Vermis_7 | 16,88 | Right-VisualAssoc (18) |
| 14 | 9,9 | -72 | -45,9 | 5,59 | 0,0000 | 637 | 0,00 | Cerebelum_8_R | 31,62 | Right-VisualAssoc (18) |
| 15 | 33,3 | -86,4 | -14,4 | 5,38 | 0,0000 | 4218 | 0,00 | Occipital_Inf_R | 0,00 | Right-BA19 |
| 16 | -0,9 | -3,6 | 10,8 | 5,04 | 0,0000 | 842 | 4,24 | Thalamus_L | 3,00 | Left-Thalamus (50) |
| 17 | -64,8 | 18,9 | -12,6 | 5,04 | 0,0000 | 285 | 8,06 | Temporal_Pole_Sup_L | 8,54 | Left-BA38 |
| 18 | 28,8 | -36,9 | 67,5 | 5,00 | 0,0000 | 34164 | 0,00 | Postcentral_R | 0,00 | Right-PrimSensory (1) |
| 19 | 28,8 | 13,5 | 20,7 | 4,81 | 0,0000 | 6293 | 5,20 | Frontal_Inf_Tri_R | 3,16 | Right-BA44 |
| 20 | -18,9 | -97,2 | 1,8 | 4,69 | 0,0000 | 1580 | 0,00 | Occipital_Mid_L | 0,00 | Left-VisualAssoc (18) |
| 21 | -1,8 | -18 | 50,4 | 4,61 | 0,0000 | 583 | 0,00 | Supp_Motor_Area_L | 0,00 | Left-BA6 |
| 22 | -60,3 | 24,3 | -22,5 | 4,46 | 0,0000 | 454 | 6,16 | Temporal_Pole_Sup_L | 7,48 | Left-BA38 |
| 23 | 57,6 | -54,9 | 9 | 4,38 | 0,0000 | 1440 | 0,00 | Temporal_Mid_R | 0,00 | Right-Fusiform (37) |
| 24 | -65,7 | -33,3 | -3,6 | 4,31 | 0,0000 | 3340 | 0,00 | Temporal_Mid_L | 0,00 | Left-BA21 |
| 25 | 54,9 | -21,6 | 52,2 | 4,25 | 0,0000 | 549 | 0,00 | Postcentral_R | 0,00 | Right-PrimSensory (1) |
| 26 | 1,8 | 5,4 | 39,6 | 4,21 | 0,0000 | 2314 | 0,00 | Cingulum_Mid_R | 0,00 | Right-BA32 |
| 27 | 24,3 | 48,6 | 29,7 | 4,21 | 0,0000 | 3787 | 0,00 | Frontal_Mid_R | 0,00 | Right-BA9 |
| 28 | 36,9 | -4,5 | 56,7 | 4,08 | 0,0000 | 3268 | 0,00 | Frontal_Mid_R | 0,00 | Right-BA6 |
| 29 | 29,7 | -87,3 | 0 | 4,04 | 0,0001 | 1182 | 0,00 | Occipital_Inf_R | 0,00 | Right-VisualAssoc (18) |
| 30 | 5,4 | -38,7 | -48,6 | 3,95 | 0,0001 | 3371 | 3,61 | Cerebelum_9_R | 32,51 | Right-Fusiform (37) |
| 31 | -9,9 | -94,5 | -16,2 | 3,94 | 0,0001 | 996 | 0,00 | Lingual_L | 0,00 | Left-VisualAssoc (18) |
| 32 | 12,6 | 18,9 | 52,2 | 3,76 | 0,0002 | 2299 | 0,00 | Frontal_Sup_R | 1,00 | Right-BA6 |
| 33 | -18 | -91,8 | -39,6 | 3,75 | 0,0002 | 282 | 4,24 | Cerebelum_Crus2_L | 19,03 | Left-VisualAssoc (18) |
| 34 | -60,3 | -21,6 | 2,7 | 3,65 | 0,0003 | 492 | 0,00 | Temporal_Sup_L | 0,00 | Left-BA22 |
| 35 | -35,1 | -33,3 | 17,1 | 3,36 | 0,0008 | 752 | 0,00 | Rolandic_Oper_L | 0,00 | Left-BA40 |
| 36 | -37,8 | -62,1 | -45 | 3,36 | 0,0008 | 152 | 0,00 | Cerebelum_Crus2_L | 21,77 | Left-Fusiform (37) |
| 37 | -21,6 | -43,2 | -45,9 | 3,35 | 0,0008 | 354 | 0,00 | Cerebelum_9_L | 22,49 | Left-Fusiform (37) |
| 38 | 57,6 | -30,6 | 47,7 | 3,32 | 0,0009 | 687 | 0,00 | SupraMarginal_R | 0,00 | Right-BA40 |
| 39 | -61,2 | -5,4 | 6,3 | 3,30 | 0,0010 | 106 | 0,00 | Temporal_Sup_L | 0,00 | Left-PrimAuditory (41) |
| 40 | 19,8 | -7,2 | 54 | 3,25 | 0,0012 | 269 | 3,00 | Frontal_Sup_R | 3,00 | Right-BA6 |
| 41 | 2,7 | 15,3 | 19,8 | 3,20 | 0,0014 | 620 | 3,00 | Cingulum_Ant_R | 2,00 | Right-BA24 |
| 42 | 10,8 | 42,3 | 47,7 | 3,03 | 0,0024 | 1642 | 0,00 | Frontal_Sup_Medial_R | 0,00 | Right-BA8 |
| 43 | -45,9 | 2,7 | -35,1 | 2,92 | 0,0035 | 296 | 0,00 | Temporal_Inf_L | 0,00 | Left-BA38 |
| 44 | -23,4 | 6,3 | 13,5 | 2,78 | 0,0054 | 683 | 0,00 | Putamen_L | 2,45 | Left-Putamen (49) |
| 45 | -43,2 | -21,6 | 10,8 | 2,66 | 0,0079 | 301 | 0,00 | Heschl_L | 0,00 | Left-PrimAuditory (41) |
| 46 | 17,1 | -34,2 | 9 | 2,56 | 0,0104 | 175 | 0,00 | Hippocampus_R | 3,00 | Right-Thalamus (50) |
| 47 | -24,3 | -47,7 | -38,7 | -2,92 | 0,0035 | 100 | 2,83 | Cerebelum_8_L | 17,23 | Left-Fusiform (37) |
| 48 | 0,9 | -3,6 | 33,3 | -2,94 | 0,0033 | 102 | 0,00 | Cingulum_Mid_R | 0,00 | Right-BA24 |
| 49 | -23,4 | -89,1 | 28,8 | -3,02 | 0,0025 | 308 | 0,00 | Occipital_Sup_L | 0,00 | Left-BA19 |
| 50 | 3,6 | -61,2 | -39,6 | -3,02 | 0,0025 | 101 | 0,00 | Vermis_9 | 30,10 | Right-Fusiform (37) |
| 51 | 10,8 | -69,3 | -11,7 | -3,04 | 0,0024 | 230 | 0,00 | Cerebelum_6_R | 1,41 | Right-VisualAssoc (18) |
| 52 | -24,3 | 15,3 | 59,4 | -3,06 | 0,0022 | 168 | 0,00 | Frontal_Mid_L | 0,00 | Left-BA6 |
| 53 | -10,8 | -19,8 | 33,3 | -3,11 | 0,0019 | 279 | 1,41 | Cingulum_Mid_L | 1,00 | Left-BA23 |
| 54 | 32,4 | 5,4 | 13,5 | -3,24 | 0,0012 | 656 | 1,00 | Insula_R | 1,00 | Right-BA44 |
| 55 | -42,3 | 26,1 | 20,7 | -3,31 | 0,0009 | 352 | 0,00 | Frontal_Inf_Tri_L | 0,00 | Left-BA44 |
| 56 | 50,4 | -29,7 | 1,8 | -3,37 | 0,0008 | 197 | 0,00 | Temporal_Sup_R | 0,00 | Right-BA22 |
| 57 | -9,9 | -55,8 | -42,3 | -3,42 | 0,0006 | 194 | 0,00 | Cerebelum_9_L | 28,72 | Left-Fusiform (37) |
| 58 | -27,9 | -76,5 | 48,6 | -3,53 | 0,0004 | 452 | 0,00 | Parietal_Sup_L | 0,00 | Left-BA7 |
| 59 | -8,1 | -61,2 | -6,3 | -3,58 | 0,0003 | 143 | 0,00 | Cerebelum_4_5_L | 2,00 | Left-BA19 |
| 60 | -43,2 | -64,8 | -2,7 | -3,79 | 0,0002 | 741 | 0,00 | Occipital_Inf_L | 0,00 | Left-BA19 |
| 61 | 44,1 | -72,9 | -21,6 | -3,85 | 0,0001 | 195 | 0,00 | Cerebelum_Crus1_R | 3,46 | Right-Fusiform (37) |
| 62 | 35,1 | -71,1 | -33,3 | -3,94 | 0,0001 | 140 | 0,00 | Cerebelum_Crus1_R | 12,21 | Right-Fusiform (37) |
| 63 | -36 | -9 | -32,4 | -4,00 | 0,0001 | 243 | 0,00 | Fusiform_L | 0,00 | Left-Parahip (36) |
| 64 | 63 | 12,6 | 9 | -4,08 | 0,0000 | 118 | 0,00 | Frontal_Inf_Oper_R | 0,00 | Right-BA44 |
| 65 | 43,2 | -84,6 | 10,8 | -4,19 | 0,0000 | 390 | 0,00 | Occipital_Mid_R | 0,00 | Right-BA19 |
| 66 | 18,9 | -31,5 | -35,1 | -4,23 | 0,0000 | 1053 | 3,74 | Cerebelum_10_R | 11,79 | Right-Fusiform (37) |
| 67 | 13,5 | 55,8 | 31,5 | -4,25 | 0,0000 | 442 | 0,00 | Frontal_Sup_Medial_R | 0,00 | Right-BA9 |
| 68 | -49,5 | -9,9 | 13,5 | -4,44 | 0,0000 | 403 | 0,00 | Rolandic_Oper_L | 0,00 | Left-PrimMotor (4) |
| 69 | 1,8 | -23,4 | 17,1 | -4,56 | 0,0000 | 532 | 4,24 | Thalamus_R | 5,83 | Right-Thalamus (50) |
| 70 | -47,7 | 6,3 | -25,2 | -4,58 | 0,0000 | 287 | 0,00 | Temporal_Mid_L | 0,00 | Left-BA38 |
| 71 | -65,7 | -38,7 | 6,3 | -4,64 | 0,0000 | 274 | 0,00 | Temporal_Mid_L | 0,00 | Left-BA21 |
| 72 | -27 | -18 | 5,4 | -4,65 | 0,0000 | 6423 | 1,00 | Putamen_L | 2,83 | Left-Putamen (49) |
| 73 | 18 | -18,9 | 11,7 | -5,05 | 0,0000 | 1696 | 0,00 | Thalamus_R | 1,00 | Right-Thalamus (50) |
| 74 | -27,9 | 36,9 | 5,4 | -5,87 | 0,0000 | 2885 | 2,83 | Frontal_Inf_Tri_L | 2,45 | Left-BA45 |
| 75 | -15,3 | 36,9 | 18,9 | -5,89 | 0,0000 | 5614 | 2,00 | Cingulum_Ant_L | 2,00 | Left-BA32 |
| 76 | -19,8 | -31,5 | -30,6 | -8,92 | 0,0000 | 939 | 2,00 | Cerebelum_4_5_L | 6,78 | Left-Fusiform (37) |
