# Supplementary material for: From single scenes to extended scenarios: The role of the ventromedial prefrontal cortex in the construction of imagery-rich events
Source: PLoS One. 2026 Feb 12;21(2):e0324764. doi: 10.1371/journal.pone.0324764 (PMC12900345; doi:10.1371/journal.pone.0324764)
Supplement: S4 Table — (DOCX) [file pone.0324764.s004.docx]

S4 Table. Cluster report from the second latent variable from the mean centered task-based Seed PLS

| Clu# | X | Y | Z | BSR | p | Size (voxels) | aal,distance | Aal.label | Ba.distance | Ba.label |
| --- | --- | --- | --- | --- | --- | --- | --- | --- | --- | --- |
| 1 | -4,5 | 53,1 | -11,7 | 24,02 | 0,0000 | 6419 | 0,00 | Frontal_Med_Orb_L | 0,00 | Left-BA10 |
| 2 | 8,1 | -35,1 | -8,1 | 10,28 | 0,0000 | 47402 | 0,00 | Cerebelum_3_R | 1,00 | Right-Parahip (36) |
| 3 | 19,8 | -9,9 | -3,6 | 9,59 | 0,0000 | 6056 | 3,00 | Pallidum_R | 3,74 | Right-GlobPal (51) |
| 4 | -23,4 | -0,9 | -6,3 | 7,92 | 0,0000 | 6032 | 1,00 | Putamen_L | 1,00 | Left-Putamen (49) |
| 5 | 2,7 | -84,6 | -18,9 | 7,15 | 0,0000 | 123 | 2,00 | Cerebelum_Crus1_R | 5,00 | Right-VisualAssoc (18) |
| 6 | 60,3 | -1,8 | 38,7 | 6,75 | 0,0000 | 2263 | 0,00 | Postcentral_R | 1,00 | Right-BA6 |
| 7 | 19,8 | -39,6 | 58,5 | 6,66 | 0,0000 | 6762 | 2,83 | Postcentral_R | 0,00 | Right-SensoryAssoc (5) |
| 8 | 0,9 | 34,2 | 13,5 | 6,24 | 0,0000 | 3156 | 0,00 | Cingulum_Ant_L | 0,00 | Right-BA24 |
| 9 | 14,4 | 29,7 | -2,7 | 5,61 | 0,0000 | 2291 | 2,24 | Caudate_R | 5,39 | Right-Caudate (48) |
| 10 | -32,4 | -69,3 | -9 | 5,60 | 0,0000 | 3341 | 0,00 | Fusiform_L | 0,00 | Left-BA19 |
| 11 | 32,4 | -83,7 | -22,5 | 5,42 | 0,0000 | 839 | 0,00 | Cerebelum_Crus1_R | 3,32 | Right-BA19 |
| 12 | -19,8 | -91,8 | -7,2 | 5,31 | 0,0000 | 1290 | 0,00 | Occipital_Inf_L | 0,00 | Left-VisualAssoc (18) |
| 13 | 0 | -5,4 | 39,6 | 5,22 | 0,0000 | 2275 | 0,00 | Cingulum_Mid_L | 0,00 | Right-BA24 |
| 14 | 12,6 | -40,5 | -45 | 5,10 | 0,0000 | 696 | 0,00 | Cerebelum_9_R | 25,16 | Right-Fusiform (37) |
| 15 | -24,3 | 18,9 | 32,4 | 4,99 | 0,0000 | 929 | 4,12 | Frontal_Mid_L | 3,00 | Left-BA9 |
| 16 | 0,9 | 7,2 | 4,5 | 4,92 | 0,0000 | 340 | 5,39 | Caudate_L | 6,32 | Right-Caudate (48) |
| 17 | 13,5 | -8,1 | 29,7 | 4,84 | 0,0000 | 1232 | 4,12 | Caudate_R | 5,10 | Right-BA24 |
| 18 | -4,5 | -57,6 | 59,4 | 4,70 | 0,0000 | 573 | 0,00 | Precuneus_L | 0,00 | Left-BA7 |
| 19 | -49,5 | 13,5 | -25,2 | 4,66 | 0,0000 | 354 | 0,00 | Temporal_Pole_Mid_L | 0,00 | Left-BA38 |
| 20 | -39,6 | -63,9 | -38,7 | 4,58 | 0,0000 | 331 | 0,00 | Cerebelum_Crus2_L | 16,91 | Left-Fusiform (37) |
| 21 | 31,5 | 35,1 | 18 | 4,56 | 0,0000 | 926 | 2,24 | Frontal_Mid_R | 1,41 | Right-BA9 |
| 22 | -45 | -31,5 | -14,4 | 4,43 | 0,0000 | 221 | 0,00 | Temporal_Inf_L | 1,00 | Left-BA20 |
| 23 | -10,8 | -27,9 | 9,9 | 4,30 | 0,0000 | 332 | 0,00 | Thalamus_L | 0,00 | Left-Thalamus (50) |
| 24 | -58,5 | 28,8 | 10,8 | 4,26 | 0,0000 | 104 | 0,00 | Frontal_Inf_Tri_L | 0,00 | Left-BA45 |
| 25 | -68,4 | 4,5 | 15,3 | 4,16 | 0,0000 | 291 | 3,00 | Postcentral_L | 4,12 | Left-BA44 |
| 26 | -58,5 | -42,3 | 22,5 | 4,16 | 0,0000 | 363 | 0,00 | Temporal_Sup_L | 0,00 | Left-BA39 |
| 27 | 35,1 | 27,9 | -16,2 | 4,12 | 0,0000 | 156 | 0,00 | Frontal_Inf_Orb_R | 0,00 | Right-BA47 |
| 28 | 22,5 | -52,2 | -12,6 | 4,07 | 0,0000 | 357 | 0,00 | Fusiform_R | 0,00 | Right-Fusiform (37) |
| 29 | -42,3 | 38,7 | 14,4 | 3,99 | 0,0001 | 427 | 0,00 | Frontal_Inf_Tri_L | 0,00 | Left-BA46 |
| 30 | 21,6 | -14,4 | 63 | 3,87 | 0,0001 | 431 | 0,00 | Precentral_R | 0,00 | Right-BA6 |
| 31 | -20,7 | -25,2 | -26,1 | 3,85 | 0,0001 | 486 | 0,00 | Cerebelum_4_5_L | 0,00 | Left-Parahip (36) |
| 32 | 26,1 | 49,5 | 3,6 | 3,82 | 0,0001 | 612 | 0,00 | Frontal_Mid_R | 0,00 | Right-BA10 |
| 33 | -44,1 | -14,4 | -28,8 | 3,67 | 0,0002 | 155 | 0,00 | Temporal_Inf_L | 0,00 | Left-BA20 |
| 34 | 38,7 | -56,7 | 7,2 | 3,63 | 0,0003 | 638 | 2,24 | Temporal_Mid_R | 1,00 | Right-Fusiform (37) |
| 35 | 22,5 | -35,1 | -32,4 | 3,60 | 0,0003 | 188 | 2,83 | Cerebelum_4_5_R | 8,60 | Right-Fusiform (37) |
| 36 | 41,4 | -53,1 | -25,2 | 3,59 | 0,0003 | 114 | 0,00 | Cerebelum_6_R | 0,00 | Right-Fusiform (37) |
| 37 | 48,6 | -74,7 | 21,6 | 3,59 | 0,0003 | 137 | 0,00 | Temporal_Mid_R | 0,00 | Right-BA19 |
| 38 | -20,7 | -4,5 | 15,3 | 3,58 | 0,0003 | 164 | 2,45 | Putamen_L | 4,12 | Left-Putamen (49) |
| 39 | 57,6 | -48,6 | 9 | 3,45 | 0,0006 | 740 | 0,00 | Temporal_Mid_R | 0,00 | Right-BA39 |
| 40 | 3,6 | 63 | 12,6 | 3,41 | 0,0007 | 387 | 0,00 | Frontal_Sup_Medial_R | 0,00 | Right-BA10 |
| 41 | -54 | 41,4 | -13,5 | 3,39 | 0,0007 | 112 | 3,00 | Frontal_Inf_Orb_L | 3,00 | Left-BA47 |
| 42 | -36,9 | 32,4 | -0,9 | 3,38 | 0,0007 | 108 | 0,00 | Frontal_Inf_Tri_L | 0,00 | Left-BA47 |
| 43 | -18,9 | 30,6 | 28,8 | 3,35 | 0,0008 | 203 | 3,00 | Frontal_Sup_L | 2,00 | Left-BA8 |
| 44 | 20,7 | 2,7 | 34,2 | 3,28 | 0,0010 | 134 | 8,25 | Frontal_Inf_Oper_R | 8,94 | Right-BA6 |
| 45 | 32,4 | 18 | -9,9 | 3,19 | 0,0014 | 192 | 0,00 | Insula_R | 0,00 | Right-Insula (13) |
| 46 | -26,1 | -54,9 | -12,6 | 3,15 | 0,0017 | 213 | 0,00 | Fusiform_L | 0,00 | Left-Fusiform (37) |
| 47 | -10,8 | -39,6 | -45,9 | 3,08 | 0,0021 | 130 | 0,00 | Cerebelum_9_L | 25,79 | Left-Fusiform (37) |
| 48 | 5,4 | -32,4 | 56,7 | 3,06 | 0,0022 | 221 | 0,00 | Paracentral_Lobule_R | 0,00 | Right-PrimMotor (4) |
| 49 | 1,8 | -70,2 | -31,5 | 3,03 | 0,0025 | 118 | 0,00 | Vermis_8 | 20,90 | Right-VisualAssoc (18) |
| 50 | 39,6 | -41,4 | 13,5 | 2,91 | 0,0036 | 137 | 3,00 | Temporal_Sup_R | 3,00 | Right-BA22 |
| 51 | 52,2 | -38,7 | 35,1 | -8,09 | 0,0000 | 10117 | 0,00 | SupraMarginal_R | 0,00 | Right-BA40 |
| 52 | 54 | 16,2 | 9 | -7,59 | 0,0000 | 5543 | 0,00 | Frontal_Inf_Oper_R | 0,00 | Right-BA44 |
| 53 | -51,3 | -29,7 | 46,8 | -7,48 | 0,0000 | 39798 | 0,00 | Parietal_Inf_L | 0,00 | Left-BA40 |
| 54 | -21,6 | 44,1 | 9 | -6,90 | 0,0000 | 611 | 3,00 | Frontal_Mid_L | 1,00 | Left-BA10 |
| 55 | 53,1 | -49,5 | -6,3 | -5,51 | 0,0000 | 903 | 0,00 | Temporal_Inf_R | 0,00 | Right-Fusiform (37) |
| 56 | 38,7 | -3,6 | 41,4 | -5,14 | 0,0000 | 3290 | 1,00 | Precentral_R | 0,00 | Right-BA6 |
| 57 | -45,9 | -65,7 | -13,5 | -4,94 | 0,0000 | 1382 | 0,00 | Occipital_Inf_L | 0,00 | Left-Fusiform (37) |
| 58 | 28,8 | -32,4 | 68,4 | -4,84 | 0,0000 | 304 | 0,00 | Postcentral_R | 0,00 | Right-PrimSensory (1) |
| 59 | -45 | -27 | -6,3 | -4,75 | 0,0000 | 394 | 4,24 | Temporal_Mid_L | 3,32 | Left-BA22 |
| 60 | 34,2 | -84,6 | 18 | -4,67 | 0,0000 | 1467 | 0,00 | Occipital_Mid_R | 0,00 | Right-BA19 |
| 61 | -11,7 | -11,7 | 68,4 | -4,61 | 0,0000 | 2171 | 0,00 | Paracentral_Lobule_L | 0,00 | Left-BA6 |
| 62 | -27,9 | -18,9 | 55,8 | -4,57 | 0,0000 | 426 | 0,00 | Precentral_L | 1,00 | Left-BA6 |
| 63 | -45,9 | 29,7 | 0,9 | -4,49 | 0,0000 | 728 | 0,00 | Frontal_Inf_Tri_L | 0,00 | Left-BA45 |
| 64 | -59,4 | -19,8 | 1,8 | -4,36 | 0,0000 | 285 | 0,00 | Temporal_Mid_L | 0,00 | Left-BA22 |
| 65 | -45,9 | -4,5 | -35,1 | -4,36 | 0,0000 | 121 | 0,00 | Temporal_Inf_L | 0,00 | Left-BA20 |
| 66 | 29,7 | 50,4 | 23,4 | -4,32 | 0,0000 | 573 | 0,00 | Frontal_Mid_R | 0,00 | Right-BA10 |
| 67 | 28,8 | -15,3 | 69,3 | -4,24 | 0,0000 | 265 | 0,00 | Precentral_R | 0,00 | Right-BA6 |
| 68 | 36 | 0,9 | -21,6 | -4,23 | 0,0000 | 148 | 1,00 | Amygdala_R | 0,00 | Right-Amygdala (53) |
| 69 | 53,1 | 20,7 | -6,3 | -4,16 | 0,0000 | 481 | 0,00 | Frontal_Inf_Orb_R | 0,00 | Right-BA47 |
| 70 | 44,1 | -31,5 | -24,3 | -4,16 | 0,0000 | 128 | 0,00 | Fusiform_R | 0,00 | Right-Fusiform (37) |
| 71 | 54 | -62,1 | -17,1 | -4,15 | 0,0000 | 392 | 0,00 | Temporal_Inf_R | 0,00 | Right-Fusiform (37) |
| 72 | 51,3 | 50,4 | -6,3 | -4,12 | 0,0000 | 875 | 0,00 | Frontal_Mid_Orb_R | 2,24 | Right-BA10 |
| 73 | -15,3 | 61,2 | 21,6 | -4,11 | 0,0000 | 573 | 0,00 | Frontal_Sup_L | 0,00 | Left-BA10 |
| 74 | 32,4 | -72 | -27 | -4,07 | 0,0000 | 700 | 0,00 | Cerebelum_Crus1_R | 7,07 | Right-Fusiform (37) |
| 75 | 11,7 | -24,3 | -25,2 | -4,06 | 0,0000 | 361 | 3,61 | Cerebelum_3_R | 8,06 | Right-Parahip (36) |
| 76 | -43,2 | -73,8 | 17,1 | -3,86 | 0,0001 | 142 | 0,00 | Occipital_Mid_L | 0,00 | Left-BA19 |
| 77 | 18 | 5,4 | -16,2 | -3,84 | 0,0001 | 122 | 0,00 | Amygdala_R | 3,00 | Right-Amygdala (53) |
| 78 | 25,2 | -50,4 | 38,7 | -3,77 | 0,0002 | 772 | 4,12 | Angular_R | 2,24 | Right-BA7 |
| 79 | -17,1 | -79,2 | -27 | -3,77 | 0,0002 | 1089 | 0,00 | Cerebelum_Crus1_L | 9,95 | Left-VisualAssoc (18) |
| 80 | 36 | -68,4 | 39,6 | -3,75 | 0,0002 | 756 | 0,00 | Angular_R | 0,00 | Right-BA39 |
| 81 | 51,3 | -25,2 | -8,1 | -3,69 | 0,0002 | 344 | 0,00 | Temporal_Mid_R | 0,00 | Right-BA22 |
| 82 | -4,5 | -58,5 | 36 | -3,64 | 0,0003 | 238 | 0,00 | Precuneus_L | 0,00 | Left-BA31 |
| 83 | -30,6 | -44,1 | 25,2 | -3,49 | 0,0005 | 221 | 9,80 | Angular_L | 10,63 | Left-BA7 |
| 84 | -1,8 | -72 | -18,9 | -3,44 | 0,0006 | 547 | 0,00 | Vermis_6 | 10,05 | Left-VisualAssoc (18) |
| 85 | 27,9 | 12,6 | 45 | -3,39 | 0,0007 | 442 | 1,41 | Frontal_Mid_R | 0,00 | Right-BA8 |
| 86 | 9,9 | -26,1 | 29,7 | -3,31 | 0,0009 | 238 | 2,45 | Cingulum_Mid_R | 1,00 | Right-BA23 |
| 87 | 18 | -79,2 | 29,7 | -3,25 | 0,0012 | 438 | 0,00 | Occipital_Sup_R | 0,00 | Right-BA19 |
| 88 | -49,5 | -53,1 | -19,8 | -3,24 | 0,0012 | 118 | 0,00 | Temporal_Inf_L | 0,00 | Left-Fusiform (37) |
| 89 | 11,7 | -59,4 | 46,8 | -3,24 | 0,0012 | 123 | 0,00 | Precuneus_R | 0,00 | Right-BA7 |
| 90 | -27,9 | -85,5 | 12,6 | -3,23 | 0,0012 | 156 | 0,00 | Occipital_Mid_L | 0,00 | Left-BA19 |
| 91 | 19,8 | 59,4 | 16,2 | -3,21 | 0,0013 | 105 | 0,00 | Frontal_Sup_R | 0,00 | Right-BA10 |
| 92 | 3,6 | 38,7 | 21,6 | -3,20 | 0,0014 | 253 | 0,00 | Cingulum_Ant_R | 0,00 | Right-BA9 |
| 93 | -34,2 | 23,4 | 37,8 | -3,11 | 0,0018 | 177 | 0,00 | Frontal_Mid_L | 0,00 | Left-BA9 |
| 94 | -16,2 | -27,9 | 31,5 | -3,07 | 0,0021 | 246 | 5,10 | Cingulum_Mid_L | 3,32 | Left-BA23 |
| 95 | 54,9 | -14,4 | 34,2 | -3,07 | 0,0022 | 124 | 0,00 | Postcentral_R | 0,00 | Right-PrimSensory (1) |
| 96 | -21,6 | -78,3 | -47,7 | -2,98 | 0,0029 | 135 | 0,00 | Cerebelum_Crus2_L | 28,91 | Left-BA19 |
