# Supplementary material for: From single scenes to extended scenarios: The role of the ventromedial prefrontal cortex in the construction of imagery-rich events
Source: PLoS One. 2026 Feb 12;21(2):e0324764. doi: 10.1371/journal.pone.0324764 (PMC12900345; doi:10.1371/journal.pone.0324764)
Supplement: Attachment — Submitted filename: Response to the reviewers.pdf [file pone.0324764.s005.pdf]

Dear Reviewers,

we thank the reviewers for their thoughtful and constructive feedback, which has greatly helped us to clarify and strengthen the manuscript. We have carefully considered each comment and believe that the revisions have substantially improved the clarity, coherence, and interpretive depth of the manuscript.
Below, we provide a point-by-point response to each of the reviewer's comments. Reviewer comments are reproduced in black, followed by our detailed responses in blue. All major textual changes have been incorporated into the revised version and are highlighted accordingly.
We hope that the revised version meets the reviewers' expectations and provides a clearer account of our findings and their contribution to the literature on mental imagery and hippocampal–prefrontal interactions.

With kind regards

Cornelia McCormick
(on behalf of all co-authors)

Reviewer #1

Using ultra-high field fMRI, the authors investigated (differences in) the neural bases of the construction of objects, scenes, and scenarios. They report that a shared neural network, including vmPFC, the hippocampus, and the posterior neocortex, was engaged in all types of mental imagery. The hippocampus was mainly involved in the construction of scenes and scenarios, while the vmPFC was maximally involved in the construction of scenarios. I think this is an interesting study, carefully conducted and using a nice experimental paradigm. I enjoyed reading it throughout. I also appreciated the reporting of participants' personal strategies to approach different imagery tasks.

I have a few suggestions to improve the completeness and clarity of the paper.

There is a strong emphasis in the paper (both in the Introduction and the Discussion) on the involvement of vmPFC in the construction of scenarios, and at times it is stated that vmPFC is not involved in the construction of single scenes. The literature cited in support of this claim provides some indirect evidence (to me, not even convincing) that this is the case. On the other hand, the most stringent neuropsychological research on the involvement of vmPFC in the construction of single scenes, testing hippocampal and vmPFC patients in boundary extension, showed that vmPFC patients - like hippocampal patients - were impaired in scene construction. In that same paper, the authors found however differences between the scene construction impairments of hippocampal and vmPFC patients. In fact, even the results of this experiment do not run against the idea that vmPFC is implicated in scene construction, even though (perhaps) less than it is implicated in scenario construction. This paper should be cited and discussed: De Luca F, McCormick C, Mullally SL, Intraub H, Maguire EA, Ciaramelli E. (2018). Boundary extension is attenuated in patients with ventromedial prefrontal cortex damage. Cortex, 108, 1-12.

Response: Our emphasis of the vmPFC as primarily involved in scenario construction while minimally involved in single scene construction may have been overstated. The reviewer's point about the boundary extension research providing evidence for vmPFC contributions to scene construction has been added to the manuscript.

In addition, we would like to clarify our position: rather than arguing that vmPFC is not involved in single scene construction, our emphasis was on the relative contribution of the vmPFC across these processes. The neuropsychological evidence the reviewer cites actually supports this nuanced view. While both hippocampal and vmPFC patients showed scene construction impairments, the differences between their impairment patterns suggest distinct but complementary roles for these regions. We added this information into the manuscript and reframed our argument in terms of relative contributions rather than presence/absence. We've accordingly adapted our abstract (p.2, l. 38-43), introduction (p.3, l.66 – p.4, l. 117) and the discussion (p.27, l.902 – p.30, l.1005).

Another inappropriate citing of the literature is on p. 3, line 65. Mind-wandering is reduced in vmPFC patients BUT NOT in patients with hippocampal lesions, in whom mind-wandering is as frequent as in controls but less episodic in nature. Please correct.
Response: We thank the reviewer for pointing out the inappropriate citing. This mistake has now been corrected in the manuscript.

New paragraph:
First, previous research described that the spontaneous initiation of internal mental events (e.g., mind-wandering episodes) appeared not only reduced in vmPFC-damaged patients (Bertossi & Ciaramelli, 2016), but also their off-task thoughts related less to the future and more to the present in comparison to controls. Importantly, the presence of hippocampal lesions did not lead to a measurable decrease of mind-wandering episodes, but their episodes appeared less visual and verbal semantic in nature (Costi et al., 2018; Luelsberg et al., 2022; McCormick, Rosenthal, et al., 2018).

At the end of the Introduction, the author should sketch the study design and the predictions, including the eye movement experiment and its rationale.
Response: We thank the reviewer for pointing out that we missed a short study design description and the missing rationale for the eye-tracking experiment. We have now included this information as a new paragraph. See p.6, l. 171-185

Was any participant in the depression range/excluded for their score at the Depression scale? P. 6.

Response: We have clarified in the manuscript that no participants were excluded based on their BDI-V score.

New paragraph: No participants were excluded based on their BDI-V score.

The authors say that the stimuli were closely matched words. Given that these were not single words, maybe it would be more appropriate to call them in a different way, like verbal cues, verbal expressions, verbal labels.

Response: We agree with the reviewer's suggestion and now consistently refer to the stimuli as verbal cues throughout the manuscript.

Also, these verbal cues were matched on what? Please specify.

Response: We apologize for the lack of clarity. The verbal cues were matched on word length (see p. 8, l. 249-250). Additionally, post hoc analyses indicated that cue vividness did not differ between imagery conditions (see p. 18, l. 529 & p.20, l. 624-627).

It is stated that an independent sample of participants rated the type of imagery triggered by these "words". Which were the precise instructions given to these participants for the rating? (It is never clear to me in reading the paper how participants get to know that they have to imagine things differently depending on imagery category, see below).

Response: We apologize for the lack of clarity. We now included two paragraphs explaining the instructions in more detail for the selection of stimuli (p. 7, l. 215 – 236) and during eye-tracking/fmri (p. 9, l. 275-313).

p. 7, line 157: based on accuracy you mean classification accuracy?

Response: Yes, we included this detail into the new paragraph.

New Paragraph: Based on these ratings, we selected cues using a discrimination-accuracy criterion: only cues that consistently elicited a single imagery category above a prespecified threshold were retained.

p. 8 line 154: they completed a practice session: practice of what task? Imagery? Classification? Both?

Response: We've added a new paragraph to clarify the course of the practice session. See p. 9, l. 262-273

In the fMRI task, upon seeing the verbal cue for imagination (e.g., a busy cafè), did participants also receive the instruction that they had to imagine it as a scene (as opposed for example to a scenario?). I understand that they were instructed on different types of imagery before the task, but did they receive an instruction to imagine something as object/scene/scenarios upon seeing the verbal cue? Please clarify in the paper, or maybe show an experimental trial. Same applies to the eye tracking experiment. Again on p. 11, it is stated that in fMRI participants were instructed to visualise the stimuli. With which instructions? Just visualization with no instruction (passive viewing)?

Response: We've clarified the fmri / eye-tracking task in more detail in a new paragraph. See p. 9, l. 275-313

I do not think that reaction times in the classification task are a relevant measure, or maybe I do not clearly find what was made of this variable. I had to go back and forth several times in the paper to understand whether there also was an analysis of response times for imagery Not clear.

Response: We thank the reviewer for this helpful comment and agree that our initial description of the reaction time (RT) measure required clarification. We have now added a paragraph clarifying the rationale for including reaction times in the classification task (p.14, l. 420-423), a new paragraph in the Results section (p.18, l.533-549) and the Discussion (p.32, l. 1062-1066). We deleted the reaction times for the vividness rating.

I do not understand the difference in the results reported for the eye tracking task (p. 16, line 363) and for the scanning task (p. 17, line 405). What did I misunderstand? Please clarify and improve the readability of the section.

Response: We have changed the paragraph to improve readability. Please see p.18, l.520-527 for eye tracking task results and p.20, l. 607-622 for scanning task.

Did imagining a scenario take longer than imagining a scene or an object? Couldn't the different findings on the neural bases of imagining objects, scene, scenarios just reflect time on task?
Response: We added a paragraph to highlight that there were no differences in visualization duration between the imagery conditions.

New paragraph: Each stimulus was displayed for 10 seconds, and participants were instructed to engage in the respective imagery task throughout the entire presentation period.

There is not much/not at all discussion of the findings from the eye movement experiment or of the source memory experiment. Not much was made of these data.
Response: We agree that the discussion of the eye-tracking and source memory findings was relatively brief. These experiments were primarily included as supporting measures to confirm task engagement and stimulus differentiation, rather than as central tests of our hypotheses. Therefore, the results were reported for completeness but not discussed in depth, as our main focus lay on the neural mechanisms underlying imagery generation during fMRI.

From the PLS results I see that vmPFC was implicated in both scene construction and, perhaps more, scenario construction. Were vmPFC regions implicated in scene and scenario construction differ in the spatial coordinates? Might there be subregions of vmPFC subserving the imagination of single scenes and the temporal unfolding of scenes in a scenario?
Response: We thank the reviewer for this insightful comment. We added a short paragraph discussing possible spatial differences on p. 29, l. 980-1006.

On p. 27, line 632, again a wrong citation: Bertossi & Ciaramelli, 2016 is a study about mind-wandering, not autobiographical memory and future thinking.
Maybe you referred to other studies, for example:
Bertossi E, Tesini C, Cappelli A, Ciaramelli E (2016). Ventromedial prefrontal damage causes a pervasive impairment of episodic memory and future thinking. Neuropsychologia, 90, 12-24.

Ciaramelli, E., Anelli, F., & Frassinetti, F. (2021). An asymmetry between past and future mental time travel following vmPFC damage. Social Cognitive and Affective Neuroscience, 16, 315-25.

Response: We thank the reviewers for this helpful remark. The citation has been corrected, and the suggested studies have been added accordingly.

Reviewer #2:

Overall Evaluation

The work is well-structured, clear in its theoretical framework, and methodologically solid. The use of 7T MRI, combined with carefully controlled imagery paradigms, represents an original and significant contribution to the study of the neural processes underlying the construction of mental events. The multivariate PLS analysis and the combination of behavioral, oculomotor, and fMRI data provide a robust triangulation of the conclusions.

However, there are several aspects that could be improved to strengthen the clarity, replicability, and impact of the work.

Major points

“Scenario” is defined as a temporally extended mental event, but at times it seems to overlap with “autobiographical episode” in the discussion. It may be helpful to clarify explicitly to what extent the scenarios are or are not autobiographical. Why was it important that the scenarios constructed by participants were not autobiographical episodes? From which perspective were the scenes and scenarios imagined (first or third person)?

Response: We appreciate the reviewer’s thoughtful comment regarding the potential overlap between scenario imagery and autobiographical episodic recall. We acknowledge that mental imagery and autobiographical memory inherently share cognitive and neural processes, such as scene construction. Thus, while participants were instructed to imagine novel scenarios rather than recall specific past events, we cannot exclude the possibility that some imagery trials involved autobiographical elements. Therefore, the precise degree to which autobiographical recall contributed to participants’ mental simulations cannot be determined. However, by instructing participants to construct novel objects, scenes or events, we wanted to minimize inter-individual variability related to personal memories and to isolate the cognitive and neural mechanisms underlying scene and scenario construction, independent of autobiographical content.

Importantly, our design did not include explicit instructions regarding the perspective (first- or third-person) to be taken, nor did we assess the perspective adopted during

imagery. We have clarified these points in the revised Discussion on p.33, l. 1093-110.

The manuscript states: “Extensive training was given to ensure that participants understood these instructions correctly and were able to differentiate between the three imagery conditions.” It would be useful to describe in more detail how this training was conducted.

Response: We apologize for the lack of clarity. We have now included a more detailed description and replaced the broad term “extensive” training with the concrete training steps. See p. 9, l. 262-273.

Sample size: N = 19 is relatively small for an fMRI study, although power may be increased by the 7T resolution and PLS analysis. It would be useful to discuss the limits of generalizability and possible interindividual variability.

Response: We agree that discussing the implications for generalizability is crucial and have added a dedicated paragraph to address this limitation in our revised manuscript on p. 33, l. 1081-1092

Were participants excluded only in the “Eye-tracking data analysis”? The text reports that 22 data points were removed; it would be helpful to specify how many participants in total were excluded. In repeated-measures ANOVA, if a data point is removed, the entire participant must typically be excluded.

Response: Thank you for this important clarification request. You are correct to point out the potential confusion in our reporting. To clarify: the "22 data points" mentioned in the eye-tracking analysis refers to individual trial-level measurements that were removed as outliers (beyond 3 standard deviations), not participants. These removals occurred across the 1,140 total trials from all 19 participants in the final sample.

Regarding participant exclusions, three participants were excluded from the entire study (not just eye-tracking) due to suboptimal performance in the classification task during scanning, reducing our sample from 22 initially enrolled to 19 participants. No additional participants were excluded specifically from the eye-tracking analysis.

You raise an excellent methodological point about repeated-measures ANOVA and missing data. Traditional repeated-measures ANOVA does indeed typically require complete data, and missing data points often necessitate excluding entire participants to maintain a balanced design. However, our approach used trial-level

outlier removal within the context of repeated-measures ANOVA, which is statistically acceptable when the missing data are random outliers rather than systematic missingness. We acknowledge that we should have been more explicit about our statistical handling of these missing trials and will clarify this in our revision. See p.15, l. 221-447.

Some effects known in the literature, such as preference for scenes over individual objects in the anterior hippocampus, were not found. This may be due to the sample size. Predefined ROIs: ROIs were selected using Neurosynth. While this is a valid approach, it would be worth discussing the risk of missing activation peaks specific to the sample (as already mentioned for the anterior hippocampus) and the implications of this.
Response: We acknowledge the reviewer's important point regarding the use of predefined ROIs selected via Neurosynth meta-analyses and added a this discussion point to the manuscript (p.30, l. 1018-1034

Control for autobiographical memory: Despite instructions to avoid personal memories, two participants reported relying on autobiographical recall. It may be useful to analyze the data with and without these participants to verify the robustness of the results. It should also be clarified how the researchers determined which participants had difficulty constructing non-autobiographical scenarios.
Response: Upon identification of participants who reported to have relied on autobiographical memories, it became evident that these participants were also the ones excluded from the experiment due to superior performance on the classification task. Therefore our PLS analysis already excludes these participants.
Regarding the identification of participants with construction difficulties, we acknowledge that our evaluation was limited to qualitative debriefing questions administered after scanning, rather than a systematic assessment during the experimental session. This represents a limitation in our methodology, as we did not implement real-time monitoring or standardized questionnaires to distinguish between autobiographical and non-autobiographical content during scenario construction.

New paragraph: “We also asked participants to describe their strategies for visualizing objects, scenes, and scenarios, and whether these strategies differed between imagery types and to which degree their constructions resembled autobiographical memories.”

Connectivity: Positive and negative connectivity in the PLS is interpreted in functional terms, but it would be useful to specify whether such patterns have been observed in similar studies and to discuss alternative interpretations.
Response: In the manuscript I changed the paragraph about negative connectivity, since the term was inaccurately used:

New paragraphs:
“Importantly, positive or negative correlations do not mean increased or decreased brain activity. The sign of the brain score / correlation reflects the polarity of a region's contribution to the latent variable rather than the direction of activation change.”
“The first LV ($p < .001$) showed a significant connectivity pattern during object ($r = -0.90$) and scene imagery ($r = -.83$), but not during scenario imagery ($r = -.14$, see **Fig. 7a**).”

It would also be interesting to test whether vividness correlates with the fMRI signal, regardless of the lack of differences between categories.
Response: We tested whether vividness covaried with fMRI signal across imagery conditions using a behavioral PLS in which, for each participant, vividness ratings were averaged within category and related to condition-specific brain activity. This analysis assessed brain–behavior covariance independent of category main effects. No latent variable reached significance ($p > 0.05$), indicating no reliable association between mean vividness and BOLD signal across conditions.

Finally, please justify why Bonferroni correction was used in some cases and Holm–Bonferroni in others, and clarify the criteria for this choice.
Response: We initially mixed Bonferroni and Holm–Bonferroni corrections across analyses. To ensure a consistent and transparent multiple-comparisons strategy, we standardized on Bonferroni for all within-ROI pairwise tests, given the small,

predefined number of contrasts per ROI and our intent to maintain a conservative familywise error control. After reapplying Bonferroni uniformly, one comparison changed its significance status; we updated the p-values in the text and revised Fig. 6 accordingly.

Results: this section is not immediately easy to follow. It would be preferable to restructure it by first reporting the significant factors and then the post hoc tests with their corresponding means.
Response: We thank the reviewer for this helpful suggestion. The Results section has been restructured to first report significant main effects, followed by post-hoc comparisons and the corresponding means to improve clarity and readability.

When reporting differences between scenes and both scenarios and objects, please also indicate whether scenarios and objects statistically differ from each other.
Response: We thank the reviewer for this suggestion. To avoid redundancy, non-significant results were not detailed in the manuscript. We have now clarified this in the Methods section

New paragraph: All planned contrasts were performed. For transparency, we report in the Results section all statistically significant findings. Non-significant contrasts are not detailed to avoid overloading the text, but were conducted and are available upon request.
Minor points:
“ni standard space”
In the Introduction (Line 63, page 3), a period is missing at the end of a sentence.
Response: This has been corrected.

The authors may consider adding a paragraph on clinical implications (e.g., in patients with vmPFC or parietal damage) and a sensitivity analysis excluding the two participants who relied on autobiographical memory.
Response: A sensitivity analysis is not required, as the two participants were already excluded from the PLS analysis based on their behavioral responses in the classification task. We have added this information to the manuscript.

New paragraph: Only two participants (which were excluded following the classification task during scanning) struggled with generating new content and reported relying on their memories in some of the trials.
