# Supplementary material for: From single scenes to extended scenarios: The role of the ventromedial prefrontal cortex in the construction of imagery-rich events
Source: PLoS One. 2026 Feb 12;21(2):e0324764. doi: 10.1371/journal.pone.0324764 (PMC12900345; doi:10.1371/journal.pone.0324764)
Supplement: Attachment — Submitted filename: Response_to_the_reviewers_auresp_3.docx [file pone.0324764.s007.docx]

Dear Reviewers,

we thank the reviewers for their thoughtful and constructive feedback, which has greatly helped us to clarify and strengthen the manuscript. Below, we provide a point-by-point response to each of the reviewer’s comments. Reviewer comments are reproduced in black, followed by our detailed responses in blue. All major textual changes have been incorporated into the revised version and are highlighted accordingly.

We hope that the revised version meets the reviewers’ expectations and provides a clearer account of our findings.

With kind regards

Cornelia McCormick

(on behalf of all co-authors)

**Reviewer #1:**

I think the authors addressed my points satisfactorily, the paper has improved very much in clarity and balance. Very interesting data the fact that different parts of vmPFC dealt with scene and scenario construction.

Page 4, line 78: there is unfortunately a typo in German and the sentence is incomplete, so please correct that so that we can assess what is stated. Please also specify the type of patients included in that research. It now reads: "and found that both patient groups were impairedKlicken oder tippen Sie hier, um Text. "

We thank the reviewer for pointing out the reference error. We have now corrected this and included the following paragraph:

“The authors also leveraged boundary extension, i.e. a cognitive process, in which viewers, after seeing a scene, automatically construct an internal representation that extends beyond the actual visual boundaries, leading to later memory for more than was shown, and found that both hippocampal and vmPFC lesions lead to impairments [16].”

Page 29: "Lesion studies have demonstrated that vmPFC patients, similar to hippocampal patients, show impairments in scene construction tasks, such as boundary extension, episodic future thinking and autobiographical memory tasks [7,8,15,54–56]". I think they refer to citation 16, not 15, as 15 is not about boundary extension.

We thank the reviewer for their careful evaluation of the references cited. We have now also included citation 16. We decided to keep citation 15 since it involves the comparison of vmPFC and hippocampal lesions during an event construction task :

“Lesion studies have demonstrated that vmPFC patients, similar to hippocampal patients, show impairments in scene construction tasks, such as boundary extension, episodic future thinking and autobiographical memory tasks [7,15,16,54–56].”
